# First person – Jillian Josimovich

**DOI:** 10.1242/bio.059142

**Published:** 2021-11-23

## Abstract

First Person is a series of interviews with the first authors of a selection of papers published in Biology Open, helping early-career researchers promote themselves alongside their papers. Jillian Josimovich is first author on ‘ Clutch may predict growth of hatchling Burmese pythons better than food availability or sex’, published in BiO. Jillian is a biologist in the lab of Dr Andrea Currylow at Geological Survey, Fort Collins Science Center, South Florida Field Station in Everglades National Park, USA, investigating applied research that can inform management efforts for imperiled species and ecosystems, with a particular focus on herpetological ecology and conservation.


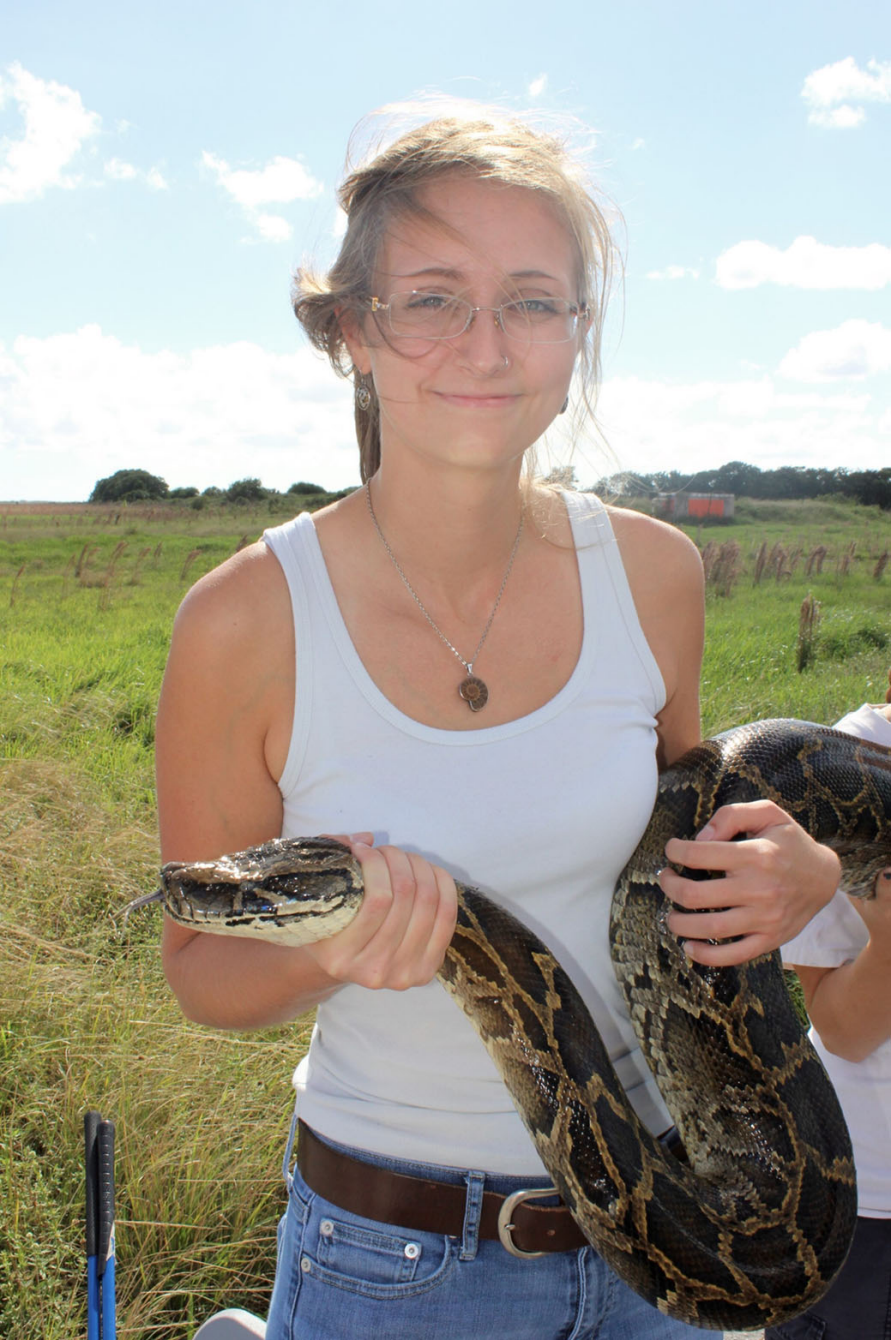


**Jillian Josimovich holding a Burmese python she captured in Everglades National Park (USGS photo by Emma Hanslowe).**

**What is your scientific background and the general focus of your lab?**

I earned my BA in biology from Vassar College in 2013 and my MS in biology from Purdue University Fort Wayne in 2018. My research background is primarily in herpetological conservation and ecology, including studies of both native and invasive reptiles. I am most interested in applied research that can inform management efforts for imperiled species and ecosystems. The USGS lab I currently work in conducts research on the prevention, control, and containment of reptiles that are invasive in the United States, such as Burmese pythons and Argentine giant tegus.

**How would you explain the main findings of your paper to non-scientific family and friends?**

This study found that hatchling Burmese pythons may grow at different rates depending on which clutch of eggs they hatched from. Their feeding behavior might also be influenced by which clutch they came from, with hatchlings from some clutches eating more than hatchlings from other clutches. This suggests that Burmese pythons from different clutches may have different survival rates in the wild because growth and behavior can influence survival.

**What are the potential implications of these results for your field of research?**

We know very little about the basic biology of Burmese pythons in their native or invasive ranges, especially hatchlings and juveniles. This research suggests that pythons from different clutches may exhibit different growth patterns, feeding behaviors, and, thereby, survival rates in the wild. Additional research could help provide insights into how clutch effects might drive fitness differences, which may be useful for informing future monitoring and management efforts for this invasive species.

**What has surprised you the most while conducting your research?**

That hatchling Burmese pythons seem to have so many different personalities. Some are much more aggressive or shy than others, which has interesting implications for their activity patterns, survival strategies, and adaptability in the wild.

“…hatchling Burmese pythons seem to have so many different personalities.”

**Figure:**
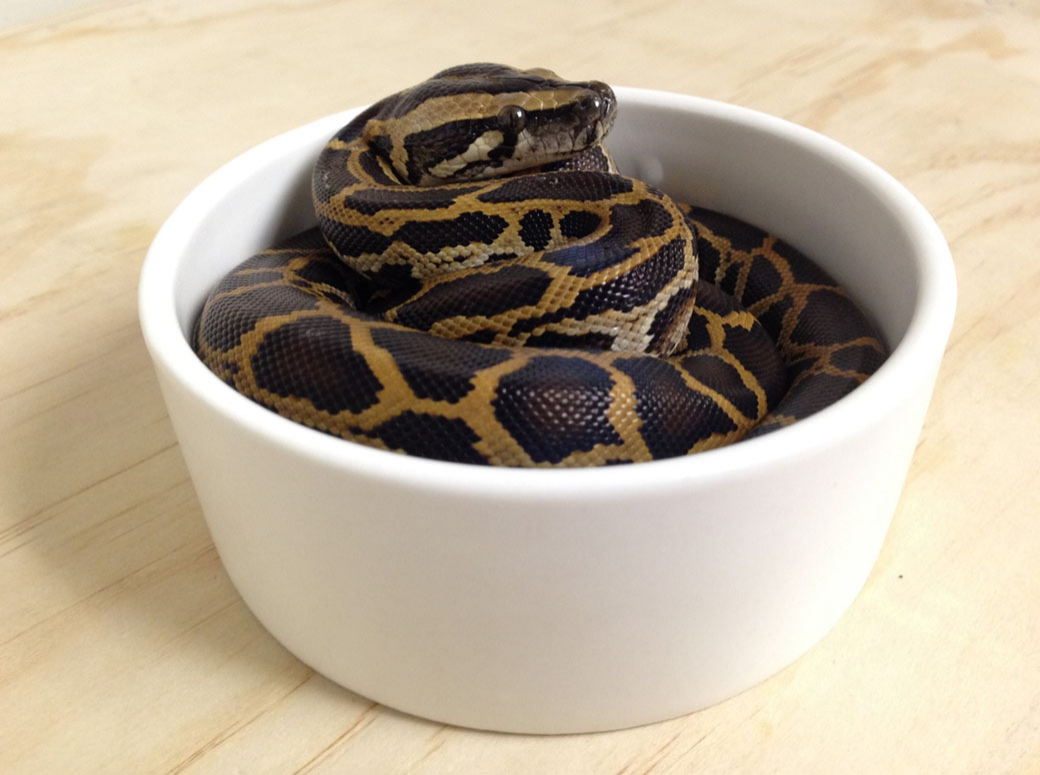
A hatchling Burmese python inside a small water dish (USGS photo by Jillian Josimovich).

**What, in your opinion, are some of the greatest achievements in your field and how has this influenced your research?**

The work done by female pioneers in invasion biology and herpetology like Dr Julie Savidge, who first brought it to scientists’ attention that brown treesnakes were causing many bird species to decline on the island of Guam in the 1980s. Many senior scientists didn't support her hypothesis and couldn't imagine that a reptile could have such devastating impacts on an entire ecosystem.

**What changes do you think could improve the professional lives of early-career scientists?**

More entry-level, paid research opportunities. It can be difficult to find paid research positions right out of college, especially in herpetology, which can be a limiting factor for some people trying to enter this field.

**What's next for you?**

I recently accepted a new biologist position working for the U.S. Fish and Wildlife Service studying threatened and endangered species such as the Florida bonneted bat, indigo snake, crested caracara, Florida scrub jay, and Florida grasshopper sparrow. I am very excited to learn more about how to manage species of conservation concern and to explore a new region of Florida!

## References

[BIO059142C1] Josimovich, J. M., Falk, B. G., Grajal-Puche, A., Hanslowe, E. B., Bartoszek, I. A., Reed, R. N. and Currylow, A. F. (2021). Clutch may predict growth of hatchling Burmese pythons better than food availability or sex. *Biology Open* 10, bio058739. 10.1242/bio.058739 34796905 PMC8609237

